# Enhanced protection in mice induced by immunization with inactivated whole viruses compare to spike protein of middle east respiratory syndrome coronavirus

**DOI:** 10.1038/s41426-018-0056-7

**Published:** 2018-04-04

**Authors:** Yao Deng, Jiaming Lan, Linlin Bao, Baoying Huang, Fei Ye, Yingzhu Chen, Yanfeng Yao, Wenling Wang, Chuan Qin, Wenjie Tan

**Affiliations:** 1 MOH Key Laboratory of Medical Virology, National Institute for Viral Disease Control and Prevention, Chinese Center for Disease Control and Prevention, Beijing, 102206 China; 2 Department of Pathogenic Biology, Hebei Medical University, Shijiazhuang, Heibei Province 050017 China; 3 Institute of Laboratory Animal Sciences, Chinese Academy of Medical Sciences (CAMS) & Comparative Medicine Center, Peking Union Medical Collage (PUMC), MOH Key Laboratory of Human Disease Comparative Medicine, Beijing, 100021 China

## Abstract

The persistent public health threat of infection with Middle East respiratory syndrome coronavirus (MERS-CoV) highlights the need for an effective and safe MERS-CoV vaccine. In this study, we prepared and vaccinated mice with either a Spike (S) protein or inactivated whole MERS-CoV (IV) with a combined adjuvant (alum+CpG) as a vaccine formulation. Similar levels of the anti-S protein IgG response and neutralizing activity were induced by both the S protein and IV vaccines. In addition, immune responses against three other structural proteins, the envelope (E), membrane (M), and nucleocapsid (N) proteins, were also detected in sera of mice that received IV. No antigen-specific T-cell immunity was detected after vaccination based on the interferon-γ ELISpot assay. Mice were transduced with Ad5-hDPP4 after the final immunization and were then challenged with MERS-CoV (1 × 10^5^ plaque-forming units). Compared with the control group (adjuvant alone), mice immunized with the S protein or IV showed slightly lower pathological damage in the lung, as well as reduced antigen expression and lung virus titers. Mice that received IV formulations also showed increased protective immunity (almost no live virus was isolated from the lung). In conclusion, our data indicate that immunization with our IV formulation induced enhanced protection in mice compared to immunization with the S protein against MERS-CoV, which should be further tested in camels and clinical trials.

## Introduction

Middle East respiratory syndrome coronavirus (MERS-CoV) was first isolated in 2012 from a patient suffering from a severe respiratory illness in Saudi Arabia^1^. As of July 2017, a total of 2040 cases in 27 countries have been reported to the World Health Organization, with 712 deaths (case fatality rate, 35%) (http://www.who.int/emergencies/mers-cov/en/). Similar to Severe acute respiratory syndrome (SARS-CoV), MERS-CoV emerged as a result of zoonotic introduction to the human population^2, 3^. Considering the ongoing MERS-CoV outbreak, it is crucial to develop intervention measures, including vaccines^4^. Currently, no licensed therapeutic treatment or vaccine is available, which highlights the urgent need for the development of an effective vaccine against MERS-CoV infection^4, 5^.

The MERS-CoV genome encodes 16 non-structural proteins (nsp1–16) and four structural proteins^2^, the spike (S), small envelope (E), membrane (M), and nucleocapsid (N) proteins. The viral structural proteins, S and N, show the highest immunogenicity^6–11^. The S protein mediates coronavirus entry into host cells by first binding to a receptor on the host-cell surface via its receptor-binding domain (RBD)^12^. Although both the S and N proteins can induce T-cell responses, neutralizing antibodies are almost solely directed against the S protein, which is the major immunodominant factor. Thus, current MERS-CoV vaccine candidates primarily use the S protein or (parts of) the gene coding for this glycoprotein^4, 5^. Vaccines against MERS-CoV infection have been developed using purified coronavirus S protein, as well as DNA or viral vector-based vaccines expressing the full-length MERS-CoV S protein or part of the S protein^13–18^. These vaccines have been tested for their ability to induce virus-neutralizing antibodies in mice or large animals, such as monkeys or camels^7, 17^. Several MERS vaccines have been developed among vaccine platforms but have been shown to confer variable degrees of immunogenicity, which necessitates the adjustment of the dose, adjuvant, and site of administration to induce optimal protective responses^4, 5, 19^. Furthermore, ongoing efforts to develop MERS-CoV vaccines should consider their immunity profiles against different antigens and correlates of protection.

An ideal MERS vaccine should induce a potent neutralizing antibody response without inducing harmful immune effects, such as virus-enhanced antibodies or immunopathology. Several previous reports relative to inactivated SARS-CoV or MERS-CoV vaccines have led to safety concerns in humans^20–26^, which are reminiscent of those reported in mice given a formalin-inactivated, whole-virus respiratory syncytial virus (RSV) vaccine and challenged with infectious RSV^27, 28^. However, preclinical evaluations of a subunit or inactivated whole-virus vaccine and Th1-type adjuvant for SARS-CoV have shown induction of serum neutralizing antibodies and protection against infection in mice challenged with an infectious virus^21^. Therefore, an appropriate adjuvant or even an adjuvant combination is required for an effective and safe vaccine formulation.

CpG oligodeoxynucleotides (namely, CpG), which are short synthetic DNA sequences consisting of unmethylated CG dinucleotides, are currently being developed as vaccine adjuvants that promote Th1-type immune responses^27^. Our previous data demonstrated the advantages of combination of two adjuvants, CpG and alum, for the induction of both Th1 and Th2 immunity in mice^15, 16, 29, 30^. The current study determined the effects of a inactivated whole MERS-CoV(IV) or S protein vaccine with a combined (alum+CpG) adjuvant on protection against MERS-CoV and the risk of lung immunopathology in mice. Furthermore, vaccination with a IV formulation containing other structural proteins (N, M, and E) than the S protein enhanced protection against MERS-CoV, as well as led to reduced viral antigen expression and pathological damage and almost no virus isolation from the lungs of mice post-challenge.

## Results

### S protein and IV formulations induced similar levels of the anti-S IgG response

**Fig. 1:**
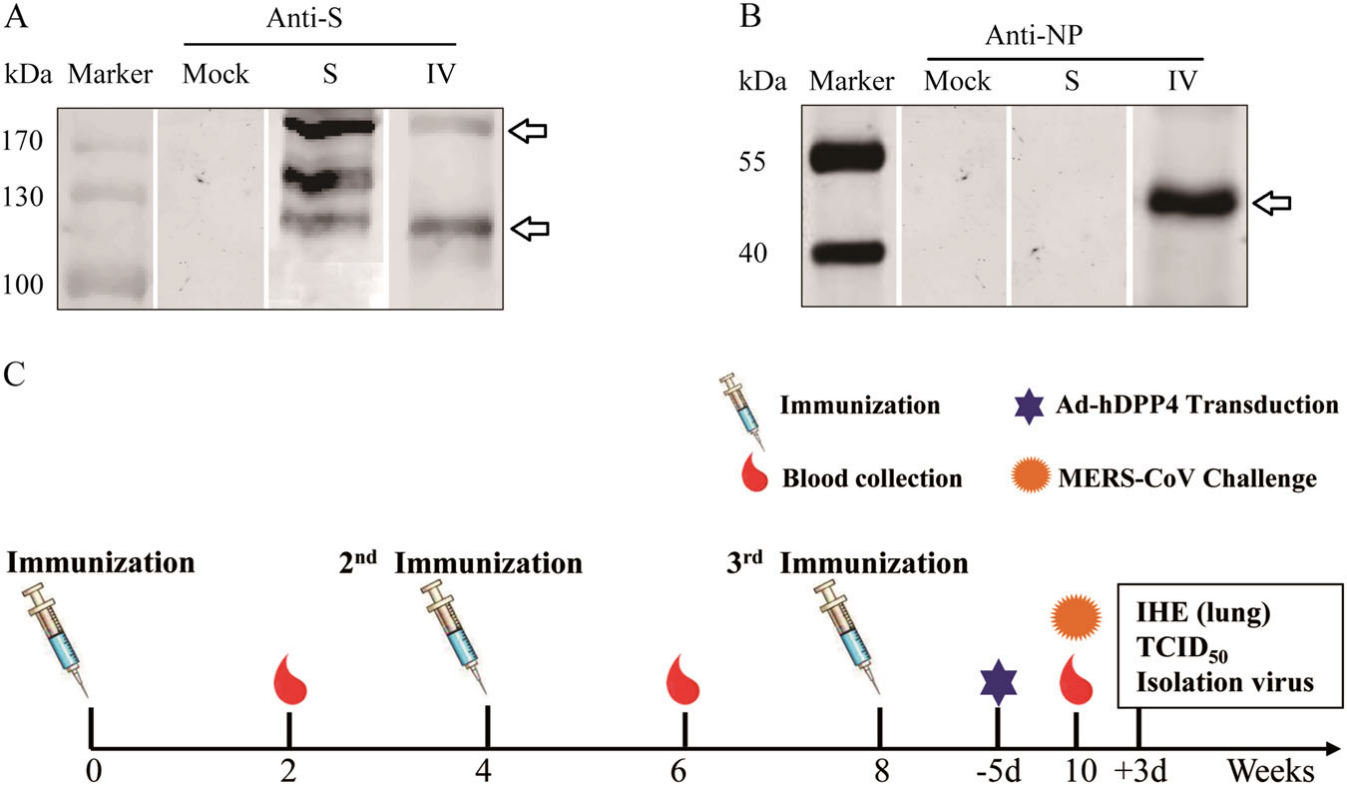
Vaccine candidates and immunization schedule. Western blot analyses of Middle East respiratory syndrome coronavirus (MERS-CoV) S and inactivated whole MERS-CoV(IV) vaccines using mouse anti-S (**a**) and anti-NP monoclonal antibodies (mAbs) (**b**). Schematic of the study (**c**)

Immunogens of the IV and S proteins were first characterized by Western blotting using anti-S (Fig. 1a) and anti-N monoclonal (Fig. 1b) antibodies produced by our laboratory^31, 32^. The S protein migrates as three polypeptides that are specifically recognized by antibodies in Western blot analysis: ~157-kDa band, which represents a monomer of the S protein ectodomain; ~110-kDa band, which represents S glycoprotein cleavage into an amino-terminal domain (S1); and ~210-kDa band, which might represent a dimer of the S1 protein^8, 18, 33^. IV migrates as two polypeptides, and the upper band represents an N-glycosylated full-length S protein and the lower band represents S1^8, 18, 33^. In addition, IV was loaded onto SDS–PAGE gels and characterized by Western blotting using anti-N mouse monoclonal antibodies that were produced by our laboratory (Fig. 1b). The results showed a 46-kDa band, which was the predicted molecular mass of IV in a previous report^32^.

**Fig. 2:**
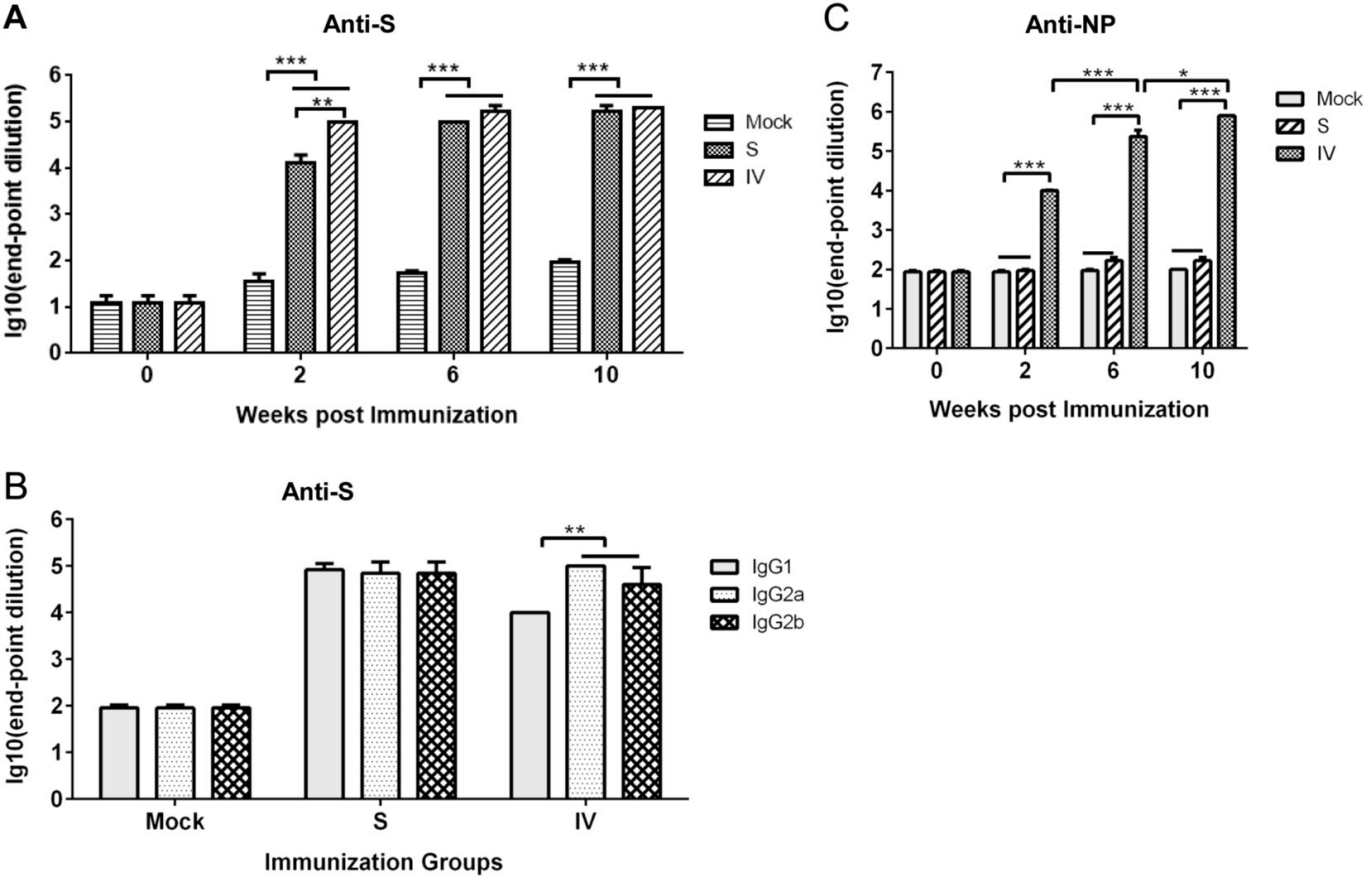
Antigen-specific IgG response after vaccination. Anti-S (**a**) and anti-NP (**b**) specific IgGs were detected at 2, 6, and 10 weeks. S protein-specific antibody isotypes induced by vaccination after 10 weeks (**c**). Values are the means ± standard error of the mean (SEM). Significant values are defined by *P < 0.05, **P < 0.01 and ***P < 0.001

To assess the immunity induced by vaccines developed from the S protein and IV and a combined adjuvant, adult female BALB/c mice (six per group) were given three i.m. immunizations at 4-week intervals of combined adjuvant alone or a formulation with either the S protein or IV at a dosage of 1 μg of S protein (Fig. 1c). After the first priming (at 2 weeks), a robust S protein-specific immunoglobulin response was detected in both the S and IV vaccine groups; the titers in mice immunized with IV were significantly higher than the titers in those immunized with the S antigen (Fig. 2a). After the second immunization, S the protein-specific IgG titers were 10^5^ at 6 weeks and did not significantly different between the S protein and IV vaccine groups (Fig. 2a). However, the S protein-specific IgG titer was not increased in either group after the third immunization.

The IgG isotypes of the S protein induced by both vaccines were tested at 10 weeks. Anti-S protein IgG1, IgG2a, and IgG2b were induced in the S protein and IV vaccine groups (Fig. 2b). The IgG2a/IgG1 and IgG2b/IgG1 ratios were ~1 (a balanced Th1- and Th2-type immune response) in the S protein vaccine group. However, the IgG2a/IgG1 and IgG2b/IgG1 ratios were >1 (a Th1-biased immune response) in the IV vaccine group.

### Specific immune responses against the N, M, and E structural proteins induced by the IV formulation

We next evaluated the N, E, and M-specific immune responses in mice that received vaccines. Significant NP-specific IgG antibodies were induced in mice 2 weeks after the first immunization with IV, but not in those that received the S protein vaccine or adjuvant alone (Fig. 2c). More robust NP-specific IgG titers reached 10^5^ dilutions of serum in mice that received the IV vaccine after the second immunization (at 6 weeks). After the third immunization, the NP-specific IgG response was further enhanced in mice that received the IV vaccine.

**Fig. 3:**
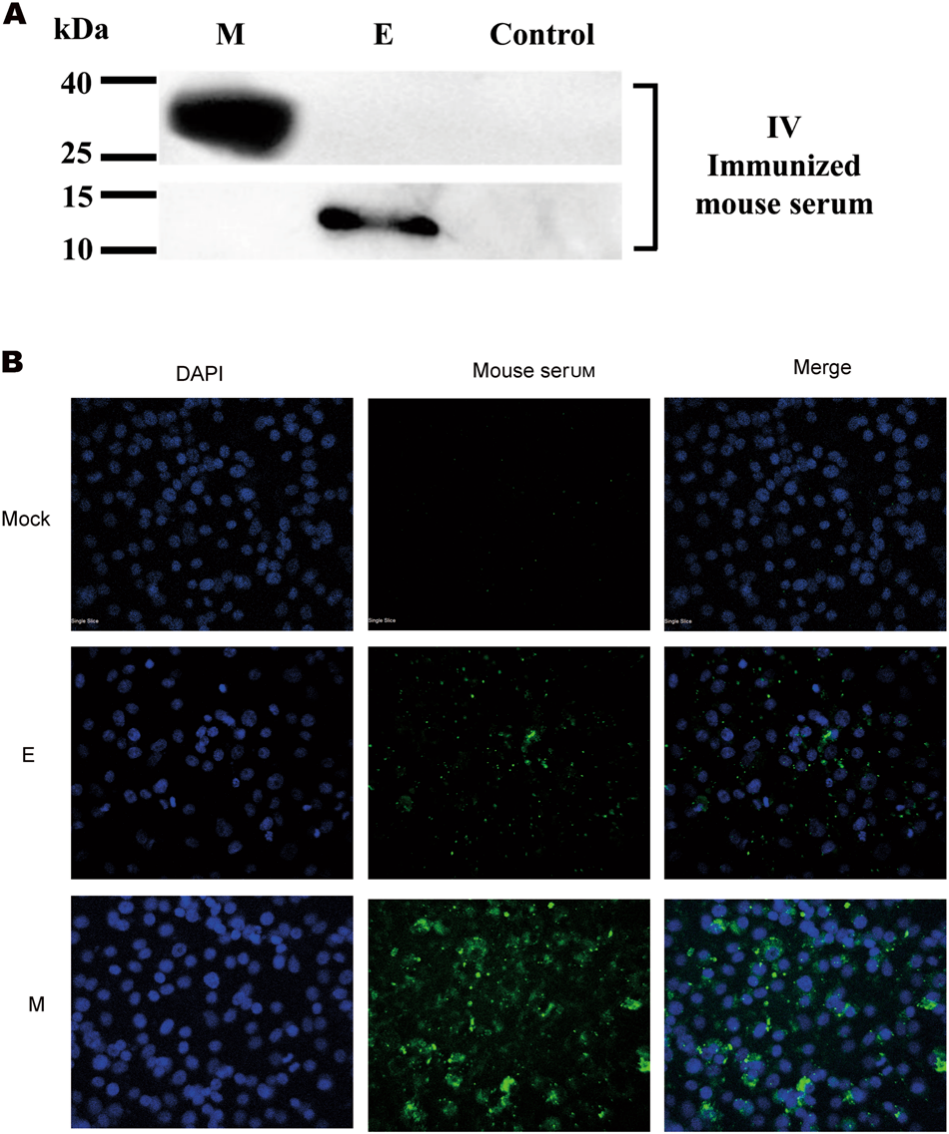
Envelope (E) and membrane (M)-specific immune responses following vaccination with IV vaccine as determined by Western blotting (WB) and indirect immunofluorescence assay (IFA). Samples were collected from 293 T cells that were transiently transfected with the pCAGGS-E (E), pCAGGS-M (M) or control plasmid pCAGGS at 24 h post-transfection and subjected to WB (**a**) or IFA (**b**) using IV-immunized mouse serum at a 1:400 dilution as the primary antibody

Samples were collected from 293 T cells that were transiently transfected with the pCAGGS-E (E), pCAGGS-M (M) or control pCAGGS plasmid at 24 h post-transfection and subjected to Western blotting (Fig. 3a) or IFA (Fig. 3b) using IV or S protein-immunized mouse serum as the primary antibody at a dilution of 1:400. E and M were detected at their predicted sizes by IV-immunized mouse serum, indicating induction of an E and M-specific immune response. Our IFA results demonstrated that specific staining for MERS-E or M protein was also observed when IV-immunized mouse serum was used as the primary antibody. However, no specific band or staining was detected using the S-immunized mouse serum as the primary antibody (data not shown).

### S protein and IV formulations induced similar levels of neutralizing activity but no detectable CMI

**Fig. 4:**
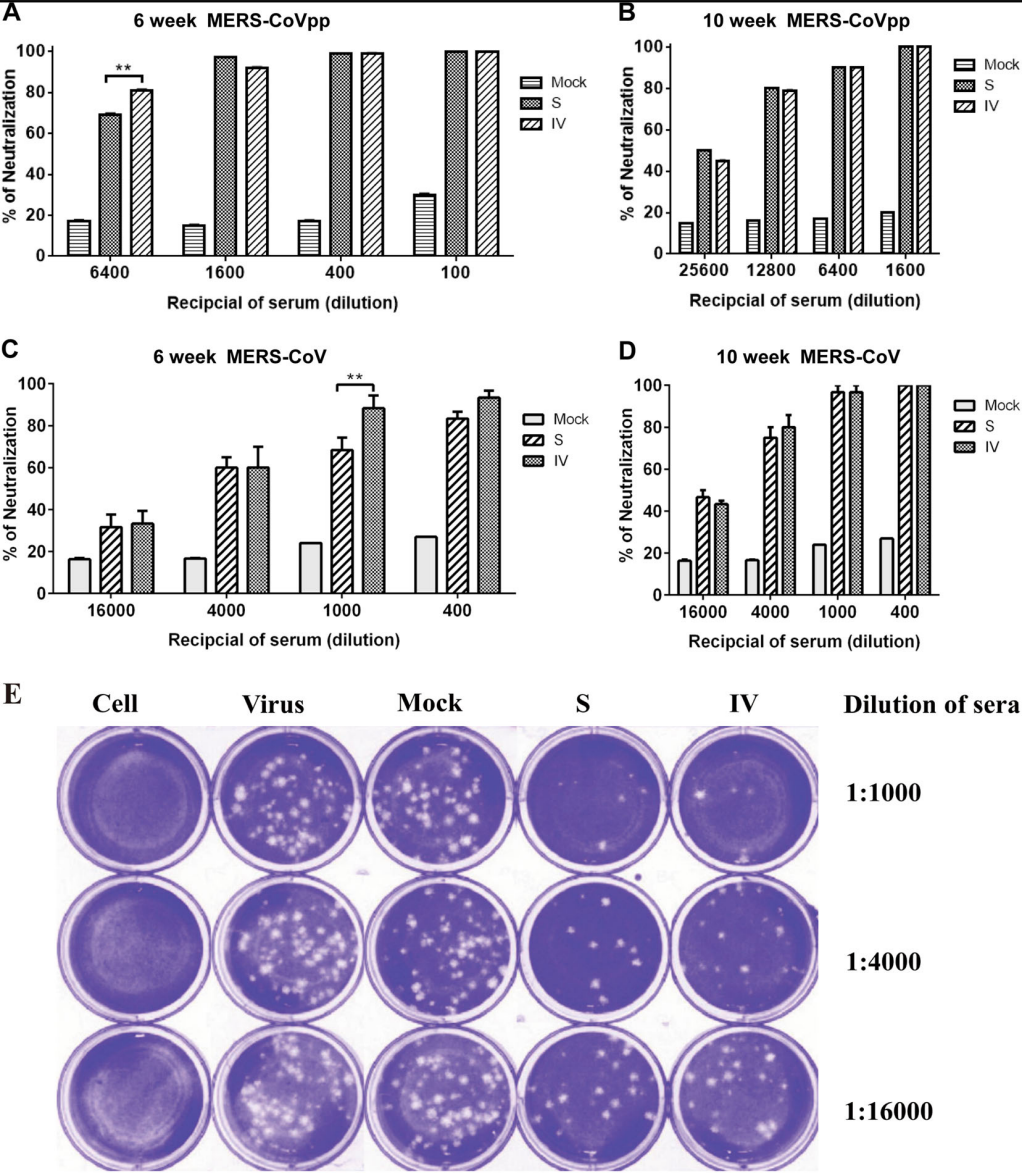
Neutralizing antibodies induced by S protein and IV vaccine against MERS-CoV pseudovirus particles and MERS-CoV. Neutralizing antibody titers against MERS-CoV pseudovirus particles. (**a**, **b**) and MERS-CoV (**c**, **d**) were determined by plaque reduction neutralization assays at 6 and 10 weeks. Representative results of the plaque reduction neutralization (PRNT) assay for the detection of neutralization activity in the sera of mice (**e**). Approximately 30 pfu of the virus stock (hCoV-EMC) was used to infect Vero cells in 12-well plates with or without heat-inactivated sera from immunized mice 2 weeks after the third immunization. PRNT50 was calculated after the plaques were counted. Significant values are defined by **p < 0.01.

Mouse sera were subjected to pseudovirus neutralization and PRNT assays after the second immunization. The serum MERS-CoV neutralization assay results largely mirrored the S protein-specific IgG results, with some notable exceptions. No serum MERS-neutralizing antibody was detectable in the mock group or other groups pre-vaccination. Robust neutralizing antibody responses were elicited by the S protein and IV combined adjuvant formulations at 6 weeks. On the basis of the pseudovirus neutralization assay, sera at a dilution of 1:6400 showed >80% neutralizing activity in the IV group, which was significantly higher than that in the S protein group (neutralizing activity ~70%; Fig. 4a). However, no significant difference in neutralizing activity in sera was found between the S protein and IV groups after the third immunization (at 10 weeks), with sera at a 1:12,800 dilution showing ~80% neutralizing activity between the S protein and IV groups (Fig. 4b). On the basis of the PRNT assay, sera at a 1:1000 dilution showed >80% neutralizing activity in the IV group at 6 weeks, which was significantly higher than that in the S protein group (neutralizing activity ~60%; Fig. 4c). No significant neutralizing activity difference in sera was found between the S protein and IV groups after the third immunization (at 10 weeks), with sera at a 1:4000 dilution showing ~ 80% neutralizing activity between the S protein and IV groups (Fig. 4d). Representative microscopy graphs of the RRNT assay are shown in Fig. 4e. As shown, the data were consistent between the pseudovirus neutralization and PRNT assay.

To evaluate CMI, mice were euthanized and splenocytes were isolated at 2 weeks after the final immunization (10 weeks). However, no significant CMI against antigens (including the S, N, M, and E proteins) were detected in any mice by the IFN-γ ELISpot assay using several synthesized 18-mer peptide libraries that overlapped with the S, E, N, and M proteins of MERS-CoV (data not shown).

### Enhanced protection of MERS-CoV was found in mice with the IV formulation

**Fig. 5:**
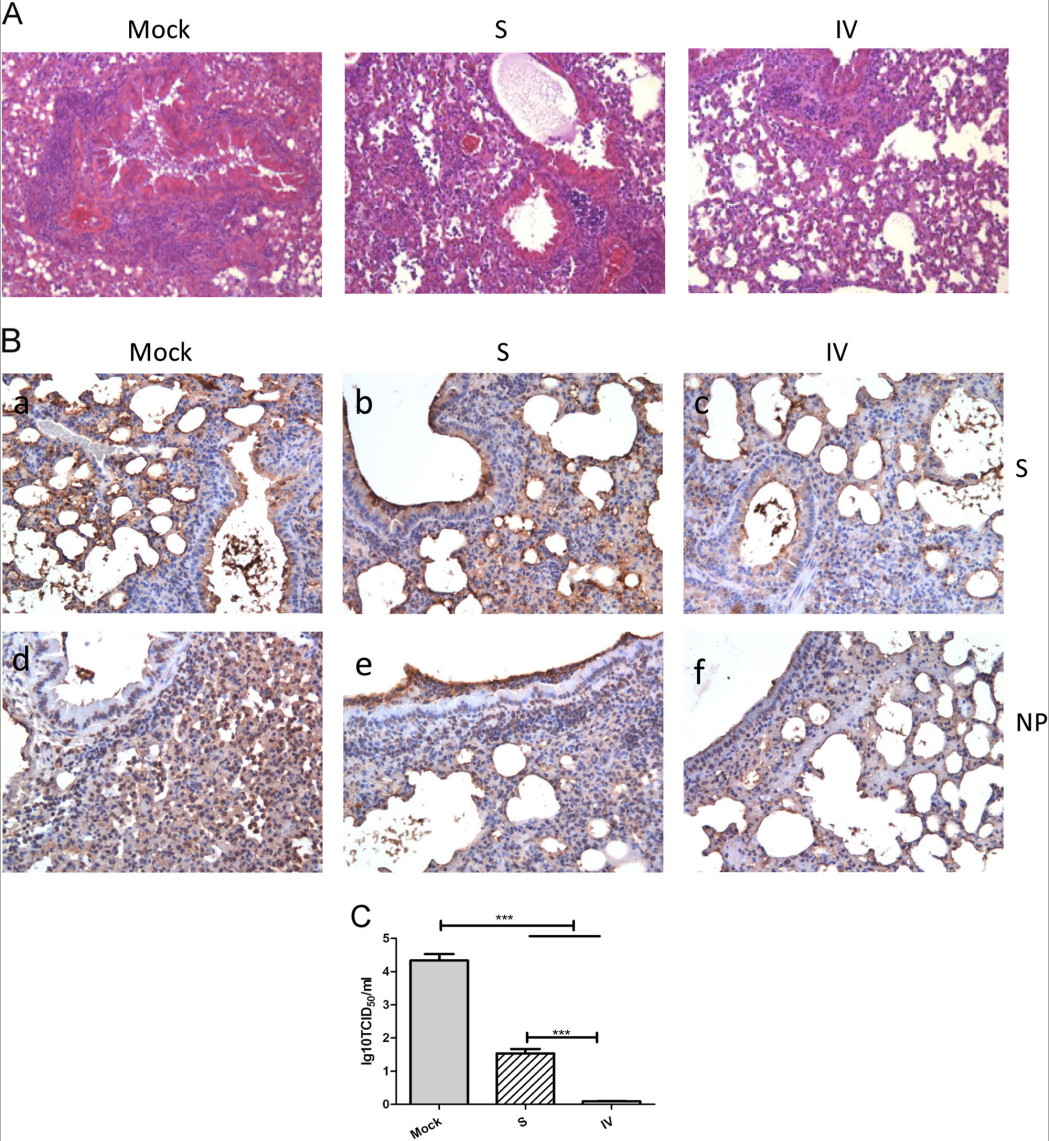
The IV formulation provides enhanced protection in mice compared to the S formulation, indicating ameliorated lung pathology, reduced viral titers and expression of virus antigens in the lungs of mice with IV or S. Representative results of hematoxylin-eosin (HE) staining (×400) in the lungs of mock-treated or immunized mice (**a**). Immunohistochemistry staining (×400) with anti-S and anti-NP mAbs (**b**). Lung virus titers 3 days after MERS-CoV challenge (**c**) as detected by virus isolation and titration at day 3 post-challenge. Values are the means ± SEM. Significant values are defined by ***P < 0.001

To assess the effect of vaccination on the mouse lung, multiple independent sites in the lung tissue samples were subjected to IE and IHC analyses at 3 days post-challenge. As shown in Fig. 5a and 5b, severe lesions including the loss of pulmonary alveoli and diffuse inflammatory cell infiltration, were detected in lung tissue of control mice. By contrast, milder lesions were observed in mice immunized with the S protein or IV, as the pulmonary alveolus was visible and infiltration of inflammatory cells was less marked. On a severity scale of 0–3 (none, mild, moderate, and severe), the adjuvant alone group was graded 3 for mononuclear cell infiltration, including lymphocytes and macrophages/monocytes, while both vaccine groups were graded 2 at 3 days post-challenge. These results indicate that S protein or IV vaccination ameliorated the respiratory tract pathology in mice after challenge with MERS-CoV (Fig. 5a). In addition, S or N protein expression was detected in lung tissue of all of the immunized groups (Fig. 5b), indicating that protection was incomplete. However, either S protein or NP expression was weaker in the lungs of mice immunized with IV compared to those immunized with S.

To assess the ability of the vaccine formulations to inhibit MERS-CoV replication in the lung, homogenized lung tissue of mice killed at day 3 post-challenge was subjected to virus isolation and titration. At day 3 post-challenge, the adjuvanted vaccine groups had significantly lower lung virus titers compared to the adjuvant-alone group. Furthermore, significant differences in the lung virus titers were detected between the two vaccine formulations at day 3 post-challenge, and the lowest lung virus titer (almost zero) was found in the group that received the IV antigen formulated with combined adjuvant (Fig. 5c). Therefore, vaccination with IV induced more effective protective immunity in mice compared to vaccination with the S protein.

## Discussion

In this study, we assessed the protective efficacy of the S protein and IV antigens of MERS-CoV against virus infection and lung immunopathology, the latter of which was previously reported to be exacerbated by alum-formulated vaccines^20^. The data suggest that S protein and IV vaccines formulated with combined adjuvant induced protective and safe immunity in mice. The enhanced protection induced by the IV vaccine containing a combined adjuvant significantly correlated with antigen-specific immunity against other structural proteins (N, M and E) compared to the vaccine formulated with the S protein of MERS-CoV. However, we also could not completely exclude the impact of the quality of the antibody response to a viral spike using recombinant protein versus those presented on inactivated virus, which need to be further clarified.

It has been reported that immunization with IV leads to lung immunopathology in mice upon challenge with live virus^20^. Therefore, inactivated MERS-CoV vaccines may present a risk of a hypersensitive-type lung pathology following a MERS-CoV infection, similar to SARS-CoV^21, 24^. Our work differs from that of Tseng et al. in several respects, including the preparation of IV (inactivated with 0.4% formaldehyde vs. gamma [γ] irradiation), adjuvant formulation (Al+CpG vs. Al or MF59), and animal model (Ad5-hDPP4 transduced mice vs. hCD26/DPP4 transgenic mice). These differences might have contributed to the different phenotypes and effects (without enhanced lung pathology vs. increased infiltrates that contained eosinophils in mice with IV) in the two studies regarding the immunity and pathology post-challenge; therefore, further work is needed.

We explored the adjuvant activity of CpG/alum combinations on the S protein and IV vaccine-induced immunity in mice. As is known, S protein or IV vaccines with alum adjuvant induce a Th2-biased response. Our data demonstrated the advantages of the combination of the two adjuvants, namely, CpG and alum, for the induction of robust antibody responses, as the S protein-specific IgG titers reached 10^5^ and a >60% neutralizing activity, which were achieved in sera at a 1:1000 dilution in both vaccine groups after the second immunization. In addition, vaccines formulated with CpG/alum combinations generated a balanced ([IgG2a+IgG2b]÷IgG1=1) or Th1-biased ([IgG2a+IgG2b]÷IgG1>1) response against the S protein in mice vaccinated with the S protein or IV, respectively. A Th1/Th2 balanced or Th1-biased immune response is generally associated with more effective control of viral infections. In Fig. 2a, mice immunized with IV produced more anti-S antibodies than those immunized with a recombinant spike protein in the 2-week post immunization group. We believe that this difference might be caused by the particle-like conformation of the S protein preserved in the immunogen of IV compared to the recombinant S protein as a soluble subunit.

The S and N proteins of CoVs are characterized by high humoral and cellular immunogenicity^34–37^. The M and E proteins are structural proteins that are anchored in the envelope of MERS-CoV particles^2^. The E protein is a small integral membrane polypeptide that forms an ion channel^38, 39^. The M protein is a typical transmembrane glycoprotein and the most abundant structural protein in CoV virions^40^. Buchholz et al.^37^ reported that the M and E proteins expressed in BHPIV3 vectors did not induce detectable resistance to a SARS-CoV challenge in hamsters. However, other reports suggest that M plays a significant role in the virus-specific humoral response and induces production of neutralizing antibodies in SARS patients^36^. In addition, the N, M, and E proteins may be antigens for antiviral cytotoxic T cells, which induce short-term resistance against a virus challenge in the absence of monoclonal antibodies in mice, as has been shown to be the case with several coronaviruses^40, 41^. In our study, the S protein and IV vaccines induced almost identical S protein antigen-specific IgG antibody responses and neutralizing activity after the last immunization. However, the antigen-specific humoral immune response against structural proteins (N, M, and E) other than the S protein was only detected in mice with IV. In addition, the level of S protein or N protein expression in the lungs of mice receiving IV was slightly lower than that in mice that received the S protein vaccine. Furthermore, a more mild pathology and lower virus titers were detected in the lungs of mice that received IV (Fig. 5), indicating that the IV formulation induced a greater protective effect in mice than the S protein formulation. Therefore, our findings indicate that this enhanced protective effect might be derived from antibodies against the N, M, and E structural proteins contained in IV.

In conclusion, the novel IV formulation (inactivated with 0.4% formaldehyde and contains the alum+CpG adjuvant) induced protective immunity but did not result in enhanced pulmonary immunopathology, which highlights the importance of the inactivation strategy and adjuvant. Interesting, although the S and IV formulations induced similar levels of the anti-S protein IgG response and neutralizing activity, the IV formulation induced a greater protective effect in mice than the S protein formulation. We suggest that immunization with the IV formulation induces enhanced protection in mice compared to the S protein against MERS-CoV, which should be further tested in camels and clinical trials.

## Materials and methods

### Ethics statement

Animal studies were conducted in strict accordance with the Guide for the Care and Use of Laboratory Animals of the People’s Republic of China. The study protocol was approved by the Committee on the Ethics of Animal Experiments of the Chinese Centre for Disease Control and Prevention (China CDC).

All experimental procedures were performed under ethyl-ether anesthesia, and every effort was made to minimize suffering. Following inoculation with MERS-CoV, all experiments were conducted in an animal biosafety level 3 (ABSL-3) facility that was constructed and accredited according to National Standard GB19489 of the Laboratory Animal Science of the Chinese Academy of Medical Sciences and Peking Union Medical College, Beijing, China.

### S Protein and inactivated MERS-CoV

Purified recombinant S protein (Sino Biological, Inc., Beijing China) was derived from baculovirus-insect cells expressing the extracellular domain of the spike protein of MERS-CoV (Human beta coronavirus 2c EMC/2012), which comprises 1289 amino acids. As described^7^, MERS-CoV (strain EMC/2012) was kindly provided by Professor Ron Fouchier (Erasmus Medical Centre, Rotterdam, The Netherlands). The virus was propagated in Vero cells (American Type Culture Collection, Manassas, VA, USA) in Dulbecco’s modified Eagle’s medium supplemented with 2% fetal calf serum, 100 international units/mL penicillin and 100 μg/mL streptomycin at 37 °C in 5% CO_2_. All experiments related to live MERS-CoV were performed according to the standard operating procedures of the biosafety level-3 (BSL-3) facility at the China CDC. Quantified MERS-CoV was inactivated with 0.4% formaldehyde for 7 days. Inactivated MERS-CoV (IV) was then centrifuged at 3000 rpm for 1 min at 4 °C, and the supernatant was collected after confirmation that it was non-infectious by titration in Vero cells. Subsequently, the supernatant was concentrated by centrifugation at 24,000 rpm for 2 h at 4 °C, and the pellet was collected and dissolved in phosphate-buffered saline (PBS) overnight. Concentrated IV was quantified using a BCA-based protein quantification kit (Applygen Technologies, Inc., Beijing, China) and stored at −80 °C until use.

### Western blotting (WB) and indirect immunofluorescence assay (IFA)

The S protein and IV were loaded onto SDS-PAGE gels and characterized by Western blotting using anti-S mouse monoclonal or anti-N monoclonal antibodies, which were produced by our laboratory^31, 32^ (Fig. 1a). To characterize the expression of the E or M protein, 293 T cells were seeded and transfected with the empty vector pCAGGS (Mock) or a plasmid encoding the MERS-CoV E or M protein, pCAGGS-E (E) or pCAGGS-M (M), respectively^42^. For IFA, cells were seeded onto glass coverslips in a 24-well plate and transfected with the indicated expression plasmids using the HD transfection reagent (Promega, Madison, WI, USA). At 24 h post-transfection, the cells were fixed in 4% formaldehyde, permeabilized in 0.5% Triton X-100, blocked in 5% BSA in PBS, and probed with primary antibodies (immunized mice serum) for 1 h at room temperature. The cells were washed with PBS and incubated for 1 h with goat anti-mouse Ig conjugated to Alexa fluor 405 at a dilution of 1:500 (Invitrogen, Shanghai, China). The cells were washed and stained with 4,6-diamidino-2-phenylindole (DAPI) (Molecular Probes) to detect nuclei. Fluorescence images were obtained and analyzed. For WB, the samples were collected at 24 h post-transfection and separated by sodium dodecyl sulfate-polyacrylamide gel electrophoresis (SDS–PAGE) for Western blotting as described previously^31, 41, 42^. IV immunized mouse serum was used as the primary antibody at a 1:400 dilution to detect the E and M proteins after vaccination with the IV formulations.

### Mice immunization and MERS-CoV challenge

The CpG ODN motif containing unmethylated cytosine preceding guanosine (5′-TCCATGACGTTCCTGACGTT-3′) was synthesized with a full phosphorothioate backbone by TAKARA BIO, Inc.). Around 6–8-week-old female BALB/c mice were divided into three groups randomly and intramuscularly (i.m.) injected with 1 μg of recombinant S protein (Sino Biological, Inc., Beijing China) or IV protein, which comprised the same dose of the S protein adjuvant with 100 μL of alum and 10 μg of CpG. For quantitative determination of the S protein of IV, a certain amount of S protein and a serial dilution of IV were loaded onto SDS–PAGE gels and characterized by Western Blotting. The S protein content in IV was evaluated by gray level screening and quantization. The control group was immunized with alum and CpG without an immunogen. The day before vaccination, the alum was mixed with the protein to a final concentration of 1 mg/mL and mixed every few hours to maintain homogeneity. As shown in Fig. 1c, mice were immunized three times at 4-week intervals. For determination of the IgG levels and neutralization activity, mice were bled by venae angularis 2 weeks after each immunization (i.e., at 2, 6, and 10 weeks). To evaluate the cell-mediated immunity (CMI), three mice from each group were euthanized and splenocytes were isolated at 2 weeks after the last immunization (10 weeks). All of the evaluated experiments were independent.

After 9 days of the last immunization, the remaining mice were lightly anesthetized with isoflurane and transduced intranasally with 2.5 × 10^8^ plaque-forming units (pfu) of Ad5-hDPP4^43^. After 5 days, transduced mice were infected intranasally with MERS-CoV (1 × 10^5^ pfu) in 50 μL of DMEM. Three days post-infection with MERS-CoV, mice were killed and their lungs were harvested. All work with MERS-CoV was conducted in ABSL-3 facilities.

### Enzyme-linked immunosorbent assay

Antigen-specific IgG antibody responses were determined by enzyme-linked immunosorbent assay (ELISA), as described previously^15, 18^. For analysis of the S protein-specific antibody isotypes, recombinant S protein was absorbed to ELISA plates and incubated overnight at 4 °C. At the same time, the recombinant NP protein^32^ expressed in prokaryotic cells and purified by a nickel column was coated on ELISA plates for analysis of NP-specific antibodies. Serum samples diluted in 1% bovine serum albumin-PBS were incubated for 2 h at room temperature (RT) and washed, followed by the addition of 100 μL of biotinylated anti-mouse IgG, IgG1, IgG2a, and IgG2b (Abcam, Cambridge, UK) conjugated to streptavidin-horseradish peroxidase (HRP; BD Biosciences, Beijing, China) and incubation for 1 h at RT. The samples were incubated with TMB (3,3′,5,5′-Tetramethylbenzidine) substrate for 10 min, and the reaction was stopped with 1 M phosphoric acid. The optical density at 450 nm (OD450) was measured using an ELISA plate reader (Molecular Devices, Sunnyvale, CA, USA).

### Pseudovirus neutralization assay

Serum neutralizing activity was determined using a MERS-CoV pseudovirus system as reported previously^15, 18^. The neutralizing antibody efficiency was calculated as: (relative luciferase units of mock sera-relative luciferase units of immune serum for a given dilution) ÷ relative luciferase units of mock sera.

### Plaque reduction neutralization assay

Plaque reduction neutralization assays (PRNT) were conducted in a BSL-3 facility, as reported previously^15, 17^. Briefly, heat-inactivated serum from immunized mice was serially diluted. After co-incubation with the same volume of MERS-CoV (hCoV-EMC), the mixtures were transferred to 12-well plates with confluent Vero cells for 1 h. Virus incubation with DMEM was used as the control. The cells were cultured at 37 °C for an additional 72 h and fixed with paraformaldehyde for 20 min. The plaques were counted after dying with gentian violet. Plaque reduction was calculated as follows: inhibition percentage = 100 × [1−(plaque number incubated with sera ÷ plaque number without sera)].

### Enzyme-linked immunospot assays

To evaluate antigen-specific T-cell responses to the vaccination regimes, interferon (IFN)-γ enzyme-linked immunospot (ELISpot) assays were performed, as described previously^15, 18^. A synthesized 18-mer peptide library that overlapped with the S, E, N, and M proteins of MERS-CoV was used. IFN-γ spot-forming cells were counted using a Bioreader (Biosys, So. Pasadena, CA).

### Hematoxylin-eosin (HE) and Immunohistochemistry(IHC)

After mice were euthanized, the lungs were collected for HE and IHC examination. Tissues were fixed using 10% neutral buffered formalin, embedded in paraffin, sectioned sequentially to a 4 μm thickness, and stained with HE prior to examination by light microscopy. For immunohistochemistry (IHC), the sections were then incubated with a rabbit-serum-derived polyclonal antibody against nucleoprotein (Sino Biological, Inc., Cat. No. 100213-RP02) or mouse-serum-derived monoclonal antibody against S protein (produced in our laboratory) at a 1:1000 dilution. The secondary antibodies were goat anti-rabbit (ZSGB-Bio, Beijing, China; Cat. No. pv-9001) and goat anti-mouse at 1:2000 dilutions. The results were evaluated by light microscopy. Multiple independent sites in the lung tissue samples were used for IHC analysis. Finally, four or five slides per animal were evaluated.

### Statistical analysis

Statistical analyses were conducted by one-way analysis of variance with Bonferroni post-test using SPSS for Windows software (ver. 17, SPSS, Inc., Chicago, IL, USA). Unpaired two-tailed Student’s *t*-test was used to compare the means between the different groups. A value of *P* < 0.05 was taken to indicate statistical significance. The results are expressed as the means ± standard deviations (s.d.). All figures were rendered using Prism 5 software (GraphPad Software, La Jolla, CA, USA).
